# New GOLD classification: longitudinal data on group assignment

**DOI:** 10.1186/1465-9921-15-3

**Published:** 2014-01-13

**Authors:** Ciro Casanova, Jose M Marin, Cristina Martinez-Gonzalez, Pilar de Lucas-Ramos, Isabel Mir-Viladrich, Borja Cosio, German Peces-Barba, Miryam Calle-Rubio, Ingrid Solanes-García, Ramón Agüero, Alfredo de Diego-Damia, Nuria Feu-Collado, Inmaculada Alfageme, Rosa Irigaray, Eva Balcells, Antonia Llunell, Juan Bautista Galdiz, Margarita Marín, Juan José Soler-Cataluña, Jose Luis Lopez-Campos, Joan B Soriano, Juan P de-Torres

**Affiliations:** 1 Pulmonary Department, Hospital Universitario Ntra. Sra. de La Candelaria, Santa Cruz de Tenerife, Tenerife, Spain; 2 Pulmonary Department, Hospital Universitario Miguel Servet, Zaragoza, Spain; 3 Pulmonary Department, Hospital Central de Asturias, Oviedo, Spain; 4 Pulmonary Department I, Hospital General Universitario Gregorio Marañón, Madrid, Spain; 5 Pulmonary Department, Hospital Son Llátzer, Palma Mallorca, Spain; 6 Pulmonary Department, Hospital Son Espases-IdISPa and CIBERES, Palma Mallorca, Spain; 7 Pulmonary Department, Fundación Jimenez Diaz, Madrid, Spain; 8 Pulmonary Department, Hospital Clinico San Carlos, Madrid, Spain; 9 Pulmonary Department, Hospital de la Santa Creu i Sant Pau, Barcelona, Spain; 10 Pulmonary Department, Hospital Marques de Valdecilla, Santander, Spain; 11 Pulmonary Department, Hospital Universitario de la Fe, Valencia, Spain; 12 Pulmonary Department, Hospital Universitario Reina Sofia. IMIBIC. UCO, Córdoba, Spain; 13 Pulmonary Department, Hospital Universitario de Valme, Sevilla, Spain; 14 Pulmonary Department, Hospital de Manacor, Manacor, Spain; 15 Pulmonary Department, Hospital del Mar and CIBERES, Barcelona, Spain; 16 Pulmonary Department, Hospital de Terrassa, Tarrasa, Spain; 17 Pulmonary Department, Hospital de Cruces, Bilbao, Spain; 18 Pulmonary Department, Hospital General de Castellon, Castellon, Spain; 19 Pulmonary Department, Hospital General de Requena, Valencia, Spain; 20 Unidad Médico-Quirúrgica de Enfermedades Respiratorias, Instituto de Biomedicina de Sevilla (IBiS), Hospital Universitario Virgen del Rocío, Sevilla, Spain; 21 Epidemiology and Clinical Research, CIMERA, Bunyola, Mallorca, Spain; 22 Pulmonary Department, Clínica Universidad de Navarra, Pamplona, Spain; 23 CIBER de Enfermedades Respiratorias (CIBERES), Instituto de Salud Carlos III, Madrid, Spain

**Keywords:** COPD, GOLD, Longitudinal

## Abstract

**Rationale:**

Little is known about the longitudinal changes associated with using the 2013 update of the multidimensional GOLD strategy for chronic obstructive pulmonary disease (COPD).

**Objective:**

To determine the COPD patient distribution of the new GOLD proposal and evaluate how this classification changes over one year compared with the previous GOLD staging based on spirometry only.

**Methods:**

We analyzed data from the CHAIN study, a multicenter observational Spanish cohort of COPD patients who are monitored annually. Categories were defined according to the proposed GOLD: FEV_1_%, mMRC dyspnea, COPD Assessment Test (CAT), Clinical COPD Questionnaire (CCQ), and exacerbations-hospitalizations. One-year follow-up information was available for all variables except CCQ data.

**Results:**

At baseline, 828 stable COPD patients were evaluated. On the basis of mMRC dyspnea versus CAT, the patients were distributed as follows: 38.2% vs. 27.2% in group A, 17.6% vs. 28.3% in group B, 15.8% vs. 12.9% in group C, and 28.4% vs. 31.6% in group D. Information was available for 526 patients at one year: 64.2% of patients remained in the same group but groups C and D show different degrees of variability. The annual progression by group was mainly associated with one-year changes in CAT scores (RR, 1.138; 95%CI: 1.074-1.206) and BODE index values (RR, 2.012; 95%CI: 1.487-2.722).

**Conclusions:**

In the new GOLD grading classification, the type of tool used to determine the level of symptoms can substantially alter the group assignment. A change in category after one year was associated with longitudinal changes in the CAT and BODE index.

## Introduction

Chronic obstructive pulmonary disease (COPD) is one of the leading causes of morbidity and mortality worldwide and is expected to increase over the coming decades [1]. The 2013 Global Initiative for Chronic Obstructive Lung Disease (GOLD) update proposed important changes to the stratification of severity in patients with COPD. These recommendations were based on the evidence that FEV_1_ is a partial descriptor of disease status. Therefore, the addition of dyspnea (modified Medical Research Council, mMRC), health status (COPD Assessment Test, CAT; Clinical COPD Questionnaire, CCQ), and exacerbations can achieve a more comprehensive assessment of COPD patients [1]. However, information on the new classification is limited because the available information on health status is based on the St George’s Respiratory Questionnaire (SGRQ), which is a surrogate marker for the CAT and no data has been published about evaluation with tools such as the CAT or CCQ [2,3]. Most importantly, the annual longitudinal progression of disease evaluated by the new GOLD proposal has not yet been explored. Recently, Agusti and colleagues described the temporal stability of the A-D groups after 3 years. However, the symptoms dimension was assessed only by the mMRC dyspnea [3,4].

Therefore, in the present study, we aimed to evaluate the distribution of patients in the CHAIN cohort, a prospective Spanish multicenter study with multidimensional evaluation of COPD patients, according to the 2013 update of the GOLD classification. We focused on the different distributions according to the tools used to evaluate the symptoms domain (mMRC, CAT, and CCQ) [3]. To determine the potential implications in clinical practice, we analyzed changes in the new GOLD classification at one year, exploring its temporal stability compared to changes in the old GOLD 2007 classification at one year.

## Methods

### Subjects

COPD patients participating in this study were part of the COPD History Assessment In SpaiN (CHAIN) cohort. CHAIN is a multicenter study of 36 prospective cohorts carried out at university hospitals in Spain [3]. COPD was defined by smoking history ≥10 pack-years and a post-bronchodilator FEV_1_/FVC <0.7 after 400 μg of inhaled albuterol. Patients were stable for at least 8 weeks and receiving optimal medical therapy. Exclusion criteria were: uncontrolled co-morbidities such as malignancy at baseline or other confounding diseases that could interfere with the study. Others methodological aspects of the study were published previously [5]. The recruitment period was January 15, 2010, to March 31, 2012 (ClinicalTrials.gov Identifier: NCT01122758). Patients are currently in the follow-up period, but the data analyzed in the present study came from the baseline and one-year follow-up appointments. December 15, 2012, was used as the cut-off date for the longitudinal data.

Briefly, at baseline and each annual visit, we evaluated anthropometric data (age, gender, and BMI), comorbidities (Charlson index; scale 0-33), smoking history, dyspnea (mMRC 0-4 scale), exacerbations during the previous year, quality of life according the Spanish versions of the CAT (scale 0-40) [6] and CCQ (scale 0-60) [7], anxiety and depression [Hospital anxiety (scale 0-21) and depression (scale 0-21) HAD scale] [8], treatments, respiratory function (arterial blood gases, spirometry, lung volume, and CO diffusion capacity), exercise capacity (six minute walking distance, 6MWD), and BODE index (scale 0-10). Data was anonymized in a database with hierarchical access control in order to guarantee secure information access. All participants signed the informed consent form previously approved by each of the ethics committee in the participating centers.

### Clinical and physiological measurements

In a personal interview, trained staff obtained the following information at the time of recruitment and at yearly appointments: age, gender, and the body mass index (BMI). BMI was calculated as the weight in kilograms divided by height in meters. A specific questionnaire was used to determine smoking status (current or former) and smoking history (pack-years). The presence of comorbidities was evaluated by the Charlson index [9].

Pulmonary function tests were performed following ATS guidelines [10]. The diffusion capacity for carbon monoxide (DLCO) was determined by the single breath technique following the ERS/ATS guidelines [11]. We have used the European Coal and Steel Community [12] predictive equations as reference values for lung function parameters. PaO_2_ was measured at rest in the sitting position while breathing room air. The 6MWD test measured the better of two walks separated by at least 30 minutes [13]. Dyspnea was evaluated by the mMRC scale [14]. The FEV_1_%, BMI, 6MWD, and MMRC values were integrated into the BODE index as previously described [15]. Exacerbations were defined by use of antibiotics, steroids, or both or admission to the hospital related to worsening respiratory symptoms. We registered the number of subjects with ≥2 exacerbations/yrs or ≥1 hospitalization/yr.

### Statistical analysis

Data are summarized as relative frequencies for categorical variables, mean and standard deviation (SD) for normally distributed scale variables, and median and 5th - 95th percentile for ordinal or non-normal scale variables. Comparisons were made between groups using Pearson chi-square, Kruskal-Wallis H test, Mann-Whitney U test, one-way ANOVA, Student t-test or Mantel-Cox test, according to the variable type and distribution. The concordance among the symptoms questionnaires was estimated by Cohen’s Kappa index. In order to determine the association between worsening GOLD category classification and changes in FEV_1_, BODE index values, and clinical parameters, we obtained ROC type-II curves and estimated the C-statistics for each one. Finally, we performed multivariate logistic regression analysis to determine the main factors at baseline associated with worsening at 12 months in the GOLD category classifications. Significance was established as a two tailed *P* < 0.05. Calculations were performed using SPSS 20.0 (Chicago, USA).

## Results

### Study population

A total of 828 patients with COPD were evaluated at baseline. The clinical and physiological characteristics of these patients are shown in Table 1. The population was mainly male (83%) and included a broad range of patients with airflow obstruction: 140 mild (16.9%), 403 moderate (48.7%), 188 severe (22.7%), and 97 very severe (11.7%). Patients reported low level of symptoms and had few hospital admissions during the previous year. Around 75% of them used inhaled muscarinic antagonists and a similar percentage used β2-agonist. In general, the patients have a normal BMI, exercise capacity, and a few comorbidities.

**Table 1:** Baseline patient characteristics

**Variable**	**Patients**
	**(N = 828)**
Gender (M/F)*	687 / 141
**Age (y)**	67 (9)
**Pack-years**	56 (28)
Active smoking*	29%
**BMI (kg/m**^**2**^**)**	28.1 (5.5)
**FEV**_**1**_**L**	1.65 (0.65)
**FEV**_**1**_**%**	56 (28)
**PaO**_**2 **_**(mmHg)**	67.4 (9.6)
**FVC L**	3.130 (0.94)
**FVC%**	88 (19)
**FEV1/FVC**	53 (11)
**6MWD (m)**	438 (104)
**Dyspnea (mMRC)†**	1 (0-4)
**BODE index†**	4 (0-6)
**IC/TLC**	0.35 (0.10)
**K**_**CO**_	74 (24)
**Charlson index†**	1 (0-5)
**Exacerbations ≥2 per patient-years**^**‡**^	0.15 (0.01)
**Hospitalization ≥1 per patient-years**^**‡**^	0.12 (0.01)
Inhaled anticholinergic*	75%
Inhaled β2-agonist*	74%
Inhaled corticosteroid*	65%
**CAT†**	11 (2-27)
**CCQ†**	1,3 (0-3.7)

Data presented as mean (SD) unless otherwise noted.

*Number and/or percent.

**†**Median (P_5-95_).

^‡^In the year before enrollment.

### Baseline distribution for the 2013 GOLD update

**Figure 1:**
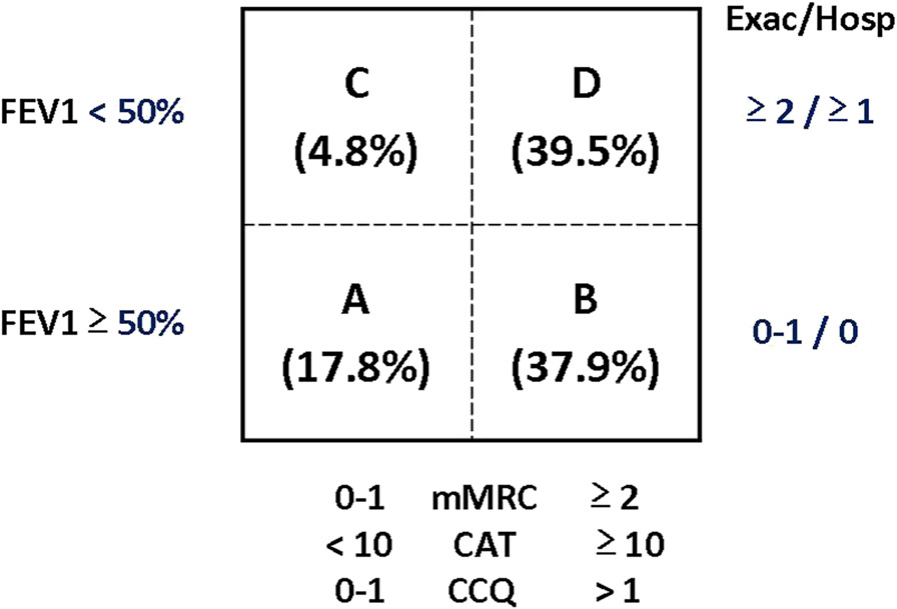
Distribution of patients in the 2013 GOLD classification using all of the symptom measurements.

Using the all scores (mMRC dyspnea, CAT, CCQ) included in the new GOLD 2013 classification to evaluate symptoms as a combined form (patients were moved to B or D score if one of these reached the cut-off point of each score: ≥2, ≥10, >1 respectively), the distribution of patients was as follows: 147 (17.8%) in group A, 314 (37.9%) in group B, 40 (4.8%) in group C, and 327 (39.5%) in group D (Figure 1). Most patients (54.1%) classified in groups C and D were categorized as such because of low FEV_1_ values, 22.4% because they had frequent exacerbation or one hospitalization during the previous year, and 23.5% because of a combination of both criteria: FEV_1_ and exacerbation during the previous year.

The clinical characteristics are shown in Table 2. A higher percentage of patients in categories A and B were actively smoking, and those in grade A were slightly younger, than those in the other groups. The patients in C and D categories walked less, had a higher BODE index and received more pulmonary pharmacological therapy.

**Table 2:** Baseline characteristics by GOLD risk groups

	**GOLD 2013**				
**Variable**	**A**	**B**	**C**	**D**	**p**
Gender (M/F)*	125 / 22	255 / 59	35 / 5	272 / 55	0.632
**Age**	66 (8)	68 (9)	68 (9)	68 (9)	0.030
**Pack-yr**	50 (24)	55 (27)	51 (28)	60 (29)	0.006
Active smoking*	40%	38%	28%	25%	0.001
**BMI (kg/m2)**	27.9 (4.7)	28.7 (6.2)	27.6 (4.6)	27.9 (5.1)	0.155
**6MWD m**	474 (97)	450 (98)	428 (93)	400 (108)	<0.001
**BODE index**^**†**^	0 (0-2)	1 (0-4)	2 (0-7)	3 (0-7)	<0.001
**Charlson index**^**†**^	1 (0-5)	1 (0-5)	1 (0-4)	1 (0-5)	0.263
Inhaled anti cholinergic*	56%	68%	87%	88%	<0.001
Inhaled β2-agonist*	53%	68%	88%	90%	<0.001
Inhaled corticosteroid*	45%	55%	82%	83%	<0.001
Theophylline*	0.7%	5%	10%	17%	<0.001

Data presented as mean (SD) unless otherwise noted.

*Number and/or percent.

†Median (P_5-95_).

**Figure 2:**
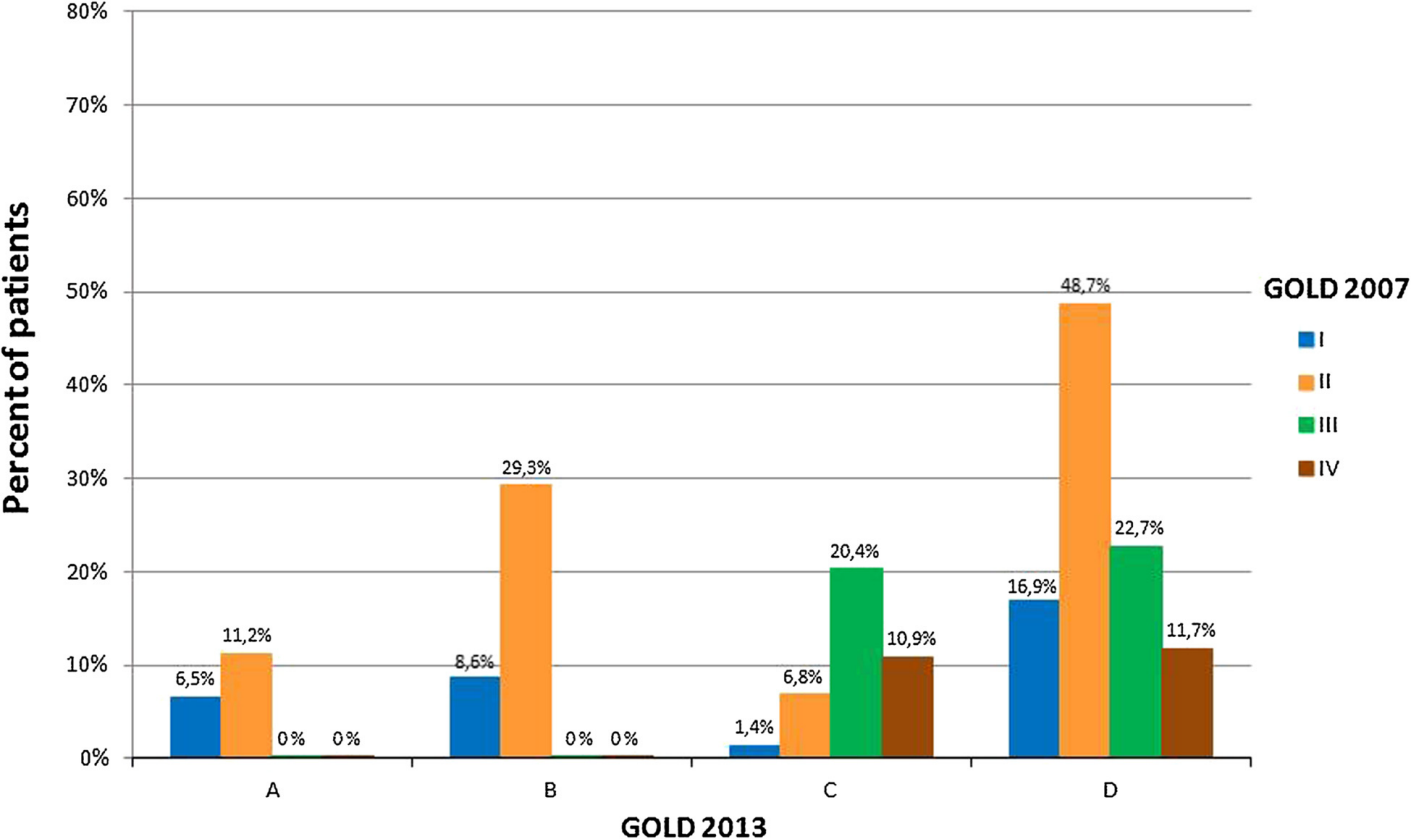
Distribution of patients with COPD at baseline according to GOLD 2007 and GOLD 2013 classification.

Figure 2 shows the classification of patients according to the 2007 (I to IV) and 2013 (A to D) GOLD classifications. The new GOLD criteria resulted in more patients being characterized into the most severe group (327 in category D) compared to the old criteria (97 in stage IV).

Category assignment was similar using the CAT and CCQ scores, but changed when the mMRC scale was used (Table 3). The largest disagreement in reassignment of patients was observed in groups A and B. According to mMRC, the percentage of patients in group B was lower and more individuals remained in group A. When a combination of these different tools was used to evaluate symptoms, changes in the distribution of patients were shown with an increased number of individuals in categories B and D (Figure 1).

**Table 3:** Tools used to evaluate 828 patients at baseline based on 2013 GOLD classification

**GOLD 2013**	**A**	**B**	**C**	**D**
**mMRC**	316 (38.2%)	146 (17.6%)	131 (15.8%)	235 (28.4%)
**CAT**	214 (27.2%)	223 (28.3%)	102 (12.9%)	249 (31.6%)
**CCQ**	220 (26.6%)	241 (29.1%)	117 (14.1%)	250 (30.2%)

The concordance between the different tools used to evaluate symptoms in GOLD 2013 classification was: mMRC and CAT, қ: 0.534, *P* < 0.001; mMRC and CCQ, қ: 0.490, *P* < 0.001; CAT and CCQ, қ: 0.673, *P* < 0.001. The concordance index among the use of one symptom score or the addition of three was low: mMRC (қ: 0.578, *P* < 0.001); CAT (қ: 0.738, *P* < 0.001); CCQ (қ: 0.747, *P* < 0.001). However, this concordance improved around 0.90 when two scores were used, regardless of the tools chosen.

No differences in comorbidities as evaluated by the Charlson index were found among categories A-D (*P* = 0.263). The proportion of patients with reported heart disease was greater in groups B and D but was not significant (A: 10.1%, B: 15.2%, C: 8.7%, D: 16.1%). However, more patients in groups B and D had HAD scores ≥11 than those in groups A and C (anxiety: A, 43%; B, 80.4%; C, 38.9%; D, 69.8%; depression: A, 21%; B, 47.8%; C, 21.1%; D, 49%; *P* < 0.001).

### Longitudinal (1 year) GOLD data

At the time of the analysis, complete information except for the CCQ was available for 526 patients at one year. Patients excluded from the longitudinal analysis showed similar baseline data for age (67.7 vs. 67.3 years, p = 0.307), gender (84% vs. 82% males, p = 0.446), level of FEV_1_ (58% vs. 60%, p = 0.140), and GOLD categories (A 18.5% vs 17.6.%, p = 0.371; B 34.3% vs 40.2%, p = 0.138; C 4.6% vs 5.2%, p = 0.709; D 43.6% vs 37.1%, p = 0.068).

**Figure 3:**
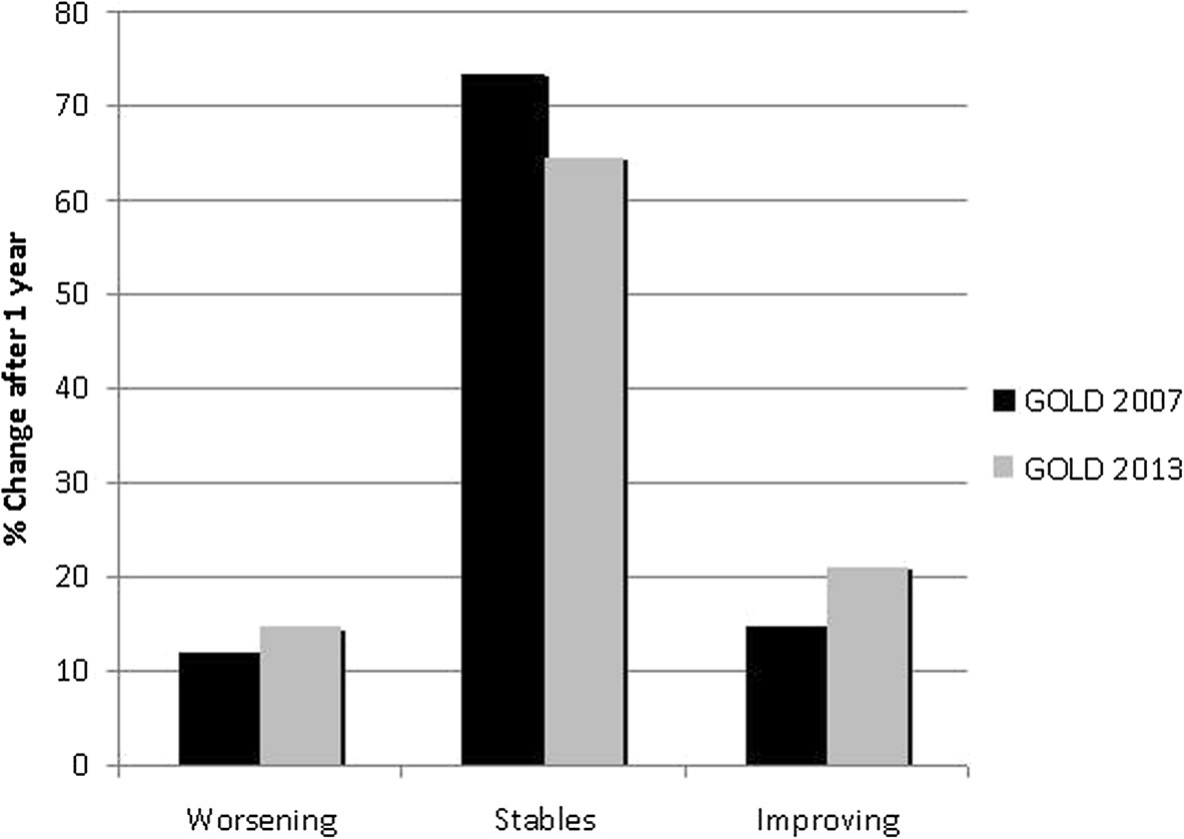
**The annual longitudinal changes by GOLD stage comparing the 2007 and 2013 versions (using mMRC dyspnea and CAT in a combined form). **We considered worsening and improving in the 2013 GOLD classification the shift from A to D and from D to A groups respectively.

**Figure 4:**
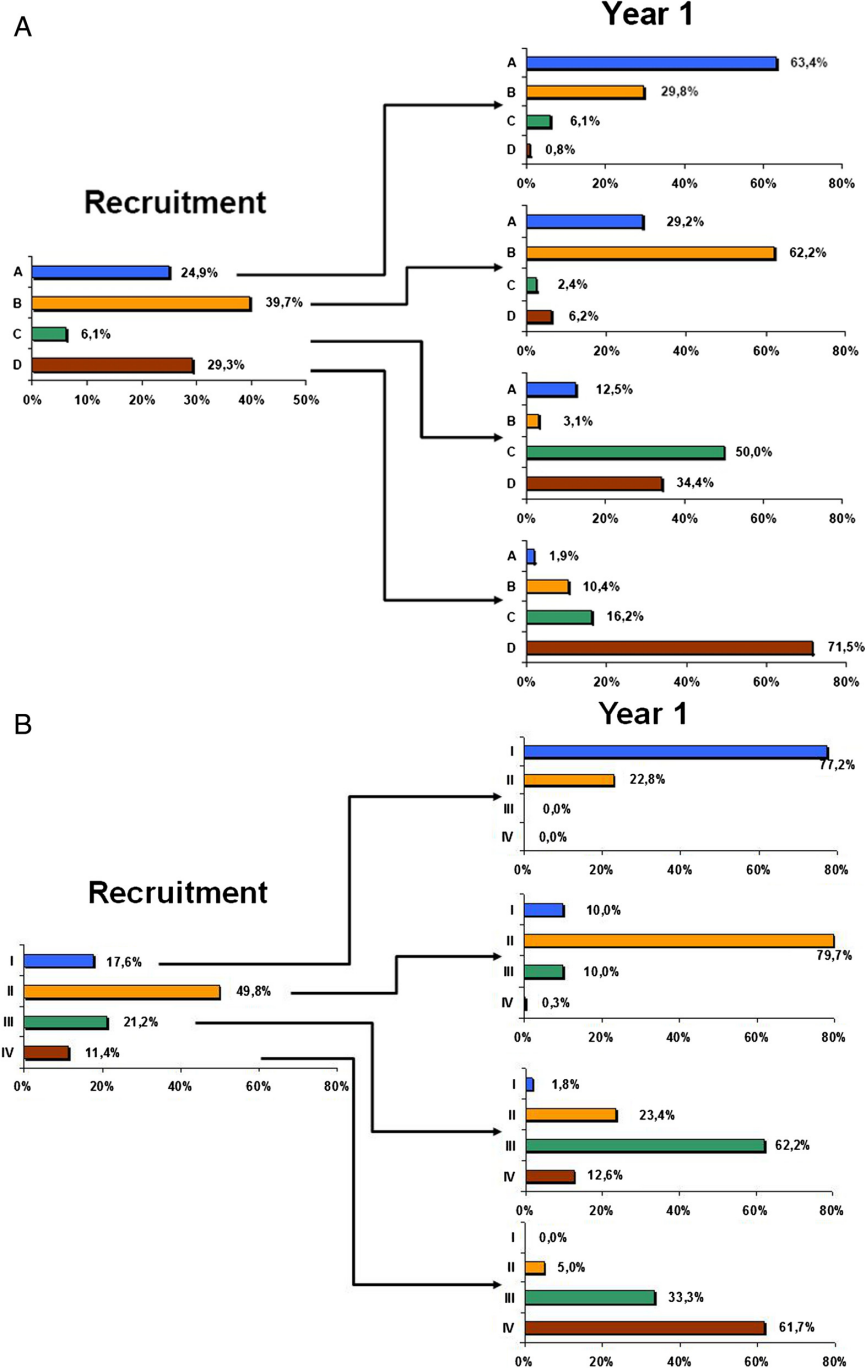
**Longitudinal changes in the CHAIN cohort. (A)**. Proportion of patients assigned to groups A-D (GOLD 2013 using mMRC dyspnea and CAT in a combined form) at baseline and the one-year follow-up. **(B)**. Proportion of patients assigned to groups I-IV (GOLD 2007) at baseline and the one-year follow-up.

Figure 3 shows the percentage of patients classified as GOLD A to D using mMRC and CAT symptoms measurements as a combined form at baseline and one year later. Longitudinal changes in the population were as follows: 64.4% of patients remained in the same category. The variability was greater for group C and lower for group D (50% and 28.6%, respectively). These annual longitudinal changes in the new GOLD classification exhibited greater variability and very low concordance compared to the old GOLD classification (қ: 0.326, *P* < 0.001; Figures 4A and 4B).

The percentage of patients experiencing ≥2 COPD exacerbations and ≥1 hospitalization during the first year were as follow respectively: 3,3% and 0% in group A, 6,7% and 0,6% in group B, 8,7% and 8,7% in group C, 12,8% and 5,2% in group D (p <0.001). The subanalysis between B and C groups only shown statistical significant differences in percent of patients with ≥1 hospitalization (p = 0.003) but not with ≥2 COPD exacerbations (p = 0.544).

The ROC analysis showed that worsening (change in grading from to any other grade: A-B, A-C, A-D, B-C, B-D, C-D) of the new GOLD stratification at one year was independently associated with longitudinal changes in the following parameters: mMRC (C-statistic 0.690, 95% CI 0.604-0.777, *P* < 0.001), CAT (C-statistic 0.716, 95% CI 0.631-0.802, *P* < 0.001), FEV_1_% (C-statistic 0.669, 95% CI 0.580-0.758, *P* < 0.001), BODE index (C-statistic 0.745, 95% CI 0.672-0.818, *P* < 0.001), and depression (C-statistic 0.608, 95% CI 0.518-0.698, *P* = 0.026). We did not find a significant association of changes in stratification with exacerbation, comorbidities, anxiety, or pulmonary inhaler treatment. The results of adjusting the logistic binary model over the potential predictors of worsening GOLD categories changes after one year are shown in Table 4. The BODE index and CAT scores remained the most important and independent predictors of changes in GOLD categories: 2.012 (95% CI: 1.487-2.722) for BODE index and 1.138 (95% CI: 1.074-1.206) for CAT.

**Table 4:** Relative risk of annual worsening of GOLD 2013 categories using univariate and multivariate logistic binary regression modeling

**Variable**	**Univariate analysis**		
	**Relative risk**	**95%CI**	**p**
**Age**	1.032	1.004-1.062	0.027
**Gender (female)**	1.542	0.761-3.122	0.229
**Active smoking**	1.086	0.637-1.853	0.761
**BMI**	1.021	0.983-1.060	0.280
**Inhaled β2-agonist**	0.824	1.487-1.393	0.470
**Inhaled anticholinergic**	0.905	0.529-1.550	0.717
**Inhaled corticosteroid**	1.065	0.639-1.774	0.809
**Charlson index**	1.047	0.898-1.220	0.557
**Anxiety**	1.021	0.967-1.077	0.450
**Depression**	1.096	1.027-1.170	0.006
FEV1%*	1.065	1.034-1.096	<0.001
mMRC*	1.956	1.494-2.562	<0.001
CAT*	1.162	1,106-1.221	<0.001
Exacerbations ≥2 or hospitalization ≥1*	0.981	0.182-5.285	0.982
BODE index*	2.136	1.664-2.742	<0.001
	**Multivariate analysis**		
	**Relative risk**	**95%CI**	**p**
**CAT**	1.138	1.074-1.206	<0.001
**BODE index**	2.012	1.487-2.722	<0.001

*The variables are included in the multivariate logistic regression model with constant using the backward stepwise method and Wald’s criteria adjusted at four iterations.

## Discussion

This observational study of COPD patients who attended pulmonary clinics has several important findings. First, we described the distribution of patients evaluated by the new 2013 GOLD classification with all of the parameters recommended by the strategy, confirming that the type of tool used to determine symptoms domain can substantially alter group assignment. Second, compared to the old 2007 GOLD classification, this new multidimensional evaluation classified a higher number of patients into more severe categories. Third, we showed that longitudinal one-year changes in groups A to D are associated with one-year changes in the CAT score and the BODE index. This novel data supports the role of symptoms and the multidimensional BODE index in the evaluation of patients with COPD. Finally, after one year of follow-up, one-third of patients changed groups; the longitudinal change was greater and had a low concordance compared to the old GOLD classification.

This study confirms that a small proportion of patients are classified into group C (low symptoms and high risk) [2], but most importantly, we confirmed that the use of different tools to evaluate symptoms (dyspnea mMRC vs. health status with the CAT or CCQ) significantly modifies grade assignment. The new GOLD strategy recommends that is unnecessary to use more than one scale for symptom evaluation. However, it is not supported by adequate scientific evidence and it is unclear whether they can be used in an additive manner [1].

Previous studies based on existing data from different cohorts recently provided information about the new GOLD classification [2,3,16-19]. All of the studies used the mMRC to evaluate symptoms and only one also used the SGRQ (as a surrogate for the CAT) to determine the patient’s grade [2]. The results were similar to those of the present study. This result is not surprising, as CAT and CCQ are questionnaires that assess several symptoms and have not demonstrated a strong correlation with the dyspnea determined by the mMRC. Han et al. suggested that potential changes can occur in the stratification of patients according to the metric used to evaluate symptoms, which our data confirmed. Importantly, we observed that the change in category assignment was greater with the CAT or CCQ compared to the SGRQ used in the previous study. In addition, we performed a novel analysis to evaluate the assignment of patients to categories if two or three symptom scores are determined in an additive form. The results showed an important shift of patients to the B and D groups, which could have implications on the recommendations for therapy. However, taking into account the concordance index, two metric symptoms appear to be enough and an adequate alternative to evaluating symptom dimensions with the new GOLD classification. Our results indicate that the best schema could include the mMRC and the CAT or CCQ. This approach captures information related to important outcomes, such as mortality with mMRC [20], avoiding disagreement and redundant data.

Similar to previous studies, more patients in our cohort were assigned to more severe stages with the new classification compared to the old classification [2,3,16-19]. However, Lange et al. showed that the prognosis of group D is worse if the patients are stratified by FEV_1_ compared to the frequency of exacerbation [15]. In our study, the number of patients categorized into this last group (D2: 22.4%) was higher than in previous studies, even one study performed in a similar clinical setting [2]. An explanation is the use of one hospitalization as the risk criteria according to the new GOLD strategy.

One of the major strengths of the present study is that it reports longitudinal data. To the best of our knowledge, we are reporting the first prospective information regarding the new GOLD A to D groups and their annual change. Approximately two-thirds of patients remained in the same category. Little differences were found by groups, though we observed greater variability for group C and lower variability for group D. This pattern shows some differences from the analysis of the ECLIPSE cohort, which also exhibited important variability in group B. However, this previous study evaluated the temporal stability after 3 years and only used the mMRC for symptom assessment [3].

Annual changes in most individuals were on the horizontal axis according to the new GOLD stratification and associated with changes in symptoms. Although one point change in the mMRC dyspnea scale is known to predicts mortality [20], no information is available on longitudinal changes in the CAT score [21].

Regarding annual changes on the vertical axis (risk) of the new GOLD approach, only a few patients changed from the A and B to the C and D categories, but 12-15% of patients in the C or D categories changed to the A or B categories. In general, changes by group were greater with the new GOLD strategy than with the old GOLD strategy, and the concordance was low. Currently, the importance of these annual changes by grade remains unknown. The clinical application of the new GOLD classification in the clinical practice remains unclear and more data with this proposed approach are needed.

Another important finding in the longitudinal changes in the new GOLD stratification is that these changes were best predicted by the BODE index. The predictive power of this index was superior to the mMRC and FEV_1_% alone, and it can be explained, in part, by a composite score such as the BODE index better integrating the changes in these variables over time.

Our study has several limitations. First, the CHAIN cohort was obtained from an observational study of patients attending pulmonary clinics and not from a general medical practice or population-based study. Therefore, the cohort might not represent the true distribution of COPD severity in the general population. However, our cohort included a broad range of disease severity, including 17% of patients in GOLD I with low mean symptom scores. Second, few women were included in the cohort, and the findings reported here cannot be extended to that gender. Nevertheless, the distribution of women into GOLD categories was similar to that of men. In addition, the main results remain unchanged when we performed a stratified subanalysis of the population by gender. Third, we have not described outcomes, such as mortality; at the time of the analysis this was not the main objective and the patients are currently being followed up.

In summary, our data based on a large cohort of well-characterized COPD patients provide important information on the assessment of patients with COPD. Using all parameters included in the new multidimensional GOLD classification, we confirmed that more patients are classified into severe categories compared to the old GOLD classification. Furthermore, we showed that the choice of tool for evaluating symptoms could alter the group assignment. According to our findings, the GOLD strategy should probably better define the thresholds by the symptoms approach, including the mMRC and CAT or CCQ. Finally, we reported the annual progression of groups A to D for the first time. The new GOLD classification is more flexible regarding category changes over time, and these changes are mainly associated with longitudinal changes in the CAT score and BODE index.

## Appendix

### CHAIN participants

***ScientificCommittee:*** Ciro Casanova (coordinator), Pilar de Lucas, Juan P. de Torres, José Luis Lopez-Campos, José María Marín, German Peces-Barba, Juan José Soler Cataluña, Joan B Soriano.

ANDALUCÍA. José Calvo Bonachera, Hospital de Torrecárdenas, Almería. Nuria Feu Collado, Hospital Universitario Reina Sofía, Córdoba. Celia Lacárcel Bautista, Hospital Ciudad de Jaén, Jaén. Adolfo Domenech, Hospital Universitario Carlos Haya, Málaga. Inmaculada Alfageme Michavila, Hospital Universitario de Valme, Sevilla.

ARAGÓN. José María Marín Trigo, Hospital Universitario Miguel Servet, Zaragoza.

ASTURIAS. Cristina Martínez González, Hospital Central de Asturias, Oviedo.

BALEARES. Rosa Irigaray, Hospital de Manacor, Manacor. Borja García-Cosío Piqueras, Hospital Son Espases, Mallorca. Isabel Mir Viladrich, Hospital Son Llátzer, Mallorca.

CANARIAS. Carlos Cabrera López, Hospital Dr. Negrín, Las Palmas de Gran Canaria. Alejandro Sánchez Acosta, Hospital Insular de Las Palmas, Las Palmas de Gran Canaria. Ciro Casanova Macario, Hospital Universitario de la Candelaria, Santa Cruz de Tenerife. Juan Abreu González, Hospital Universitario de Canarias, Santa Cruz de Tenerife.

CANTABRIA. Ramón Agüero Balbin, Hospital Marqués de Valdecilla, Santander.

CATALUÑA. Eva Balcells, Hospital del Mar, Barcelona. Elena Miguel Campos, Hospital Sant Joan Despí, Barcelona. Alicia Marin, Hospital German Trias y Pujol, Badalona, Barcelona. Ingrid Solanes García, Hospital de la Santa Creu i Sant Pau, Barcelona. Antonia Llunell Casanova, Hospital de Terrassa, Tarrasa. Amalia Moreno, Hospital Parc Taulí, Sabadell.

EXTREMADURA. Francisca Lourdes Márquez Pérez, Hospital Infanta Cristina, Badajoz. Juan Antonio Riesco Miranda, Hospital San Pedro Alcántara, Cáceres.

GALICIA. Julia Tabara Rodríguez, Hospital Juan Canalejo, La Coruña. Rafael Golpe Gómez, Hospital General Calde, Lugo.

MADRID. Germán Peces-Barba Romero, Fundación Jiménez Díaz, Madrid. Miriam Calle Rubio, Hospital Clínico San Carlos, Madrid. Javier de Miguel Díez, Hospital Gregorio Marañón, Madrid. Pilar de Lucas Ramos, Hospital Gregorio Marañón, Madrid. Francisco García Río, Hospital La Paz, Madrid. Salvador Díaz Lobato, Hospital Ramón y Cajal, Madrid.

NAVARRA. Juan Pablo de Torres, Clínica Universidad de Navarra, Pamplona.

PAÍS VASCO. Juan Bautista Galdiz Iturri, Hospital de Cruces, Bilbao.

VALENCIA. Margarita Marín Royo, Hospital General de Castellón, Castellón. Juan José Soler Cataluña, Hospital General de Requena, Requena. Alfredo de Diego Damia, Hospital Universitario La Fe, Valencia.

## Competing interests

The authors declare that they have no competing interests.

## Authors’ contributions

CC, JP-T: conception and design, recruitment of patients, analysis and interpretation and drafting the manuscript for important intellectual content. JMM, CM-G, P L-R, IM-V, BC, GP-B, MC-R, IS-G, RA, AD-D, NF-C, IA, RI, EB, AL, JBGI, MM, JJS: conception and design, recruitment of patients, analysis and interpretation. JLL-C, JBS: conception and design, analysis and interpretation and drafting the manuscript for important intellectual content. All authors read and approved the final manuscript.
