# Erratum: New GOLD classification: longitudinal data on group assignment

**DOI:** 10.1186/s12931-014-0140-z

**Published:** 2014-12-11

**Authors:** Ciro Casanova, Jose M Marin, Cristina Martinez-Gonzalez, Pilar de Lucas-Ramos, Isabel Mir-Viladrich, Borja Cosio, German Peces-Barba, Miryam Calle-Rubio, Ingrid Solanes-García, Ramón Agüero, Alfredo de Diego-Damia, Nuria Feu-Collado, Inmaculada Alfageme, Rosa Irigaray, Eva Balcells, Antonia Llunel, Juan Bautista Galdiz-Iturri, Margarita Marín, Juan José Soler, Jose Luis Lopez-Campos, Joan B Soriano, Juan P de-Torres, the COPD History Assessment In SpaiN (CHAIN) cohort

**Affiliations:** Pulmonary Department, Hospital Universitario Ntra. Sra. de La Candelaria, Carretera del Rosario n° 145, 38010 Santa Cruz de Tenerife, Spain; Pulmonary Department, Hospital Universitario Miguel Servet, Zaragoza, Spain; Pulmonary Department, Hospital Central de Asturias, Oviedo, Spain; Pulmonary Department I, Hospital Gregorio Marañón, Madrid, Spain; Pulmonary Department, Hospital Son Llátzer, Mallorca, Spain; Pulmonary Department, Hospital Son Dureta, Mallorca, Spain; Pulmonary Department, Fundación Jimenez Diaz, Madrid, Spain; Pulmonary Department, Hospital Clinico San Carlos, Madrid, Spain; Pulmonary Department, Hospital San Pablo y la Santa Cruz, Barcelona, Spain; Pulmonary Department, Hospital Marques de Valdecilla, Santander, Spain; Pulmonary Department, Hospital Universitario de la Fe, Valencia, Spain; Pulmonary Department, Hospital Universitario Reina Sofía, Córdoba, Spain; Pulmonary Department, Hospital Universitario de Valme, Sevilla, Spain; Pulmonary Department, Hospital de Manacor, Mallorca, Spain; Pulmonary Department, Hospital del Mar, Barcelona, Spain; Pulmonary Department, Hospital de Tarrasa, Tarrasa, Spain; Pulmonary Department, Hospital de Cruces, Bilbao, Spain; Pulmonary Department, Hospital General de Castellon, Castellon, Spain; Pulmonary Department, Hospital General de Requena, Valencia, Spain; Unidad Médico-Quirúrgica de Enfermedades Respiratorias. Instituto de Biomedicina de Sevilla (IBiS), Hospital Universitario Virgen del Rocío, Sevilla, Spain; Epidemiology and Clinical Research, CIMERA, Bunyola, Mallorca, Spain; Pulmonary Department, Clínica Universitaria de Navarra, Pamplona, Spain; CIBER de Enfermedades Respiratorias (CIBERES), Instituto de Salud Carlos III, Madrid, Spain

## Erratum

After publication of this work [1], regrettably we noted that there were two errors in the illustrations, which were added when the paper was under revision and inadequately checked.

In Table 1, when describing the BODE data, the median value was 2, so instead of BODE index† 4 (0-6), the correct one is BODE index† 2 (0-6).

Figure two was plotted with incorrect percentages. The correct Figure two is shown next (see Figure 1).

**Figure 1:**
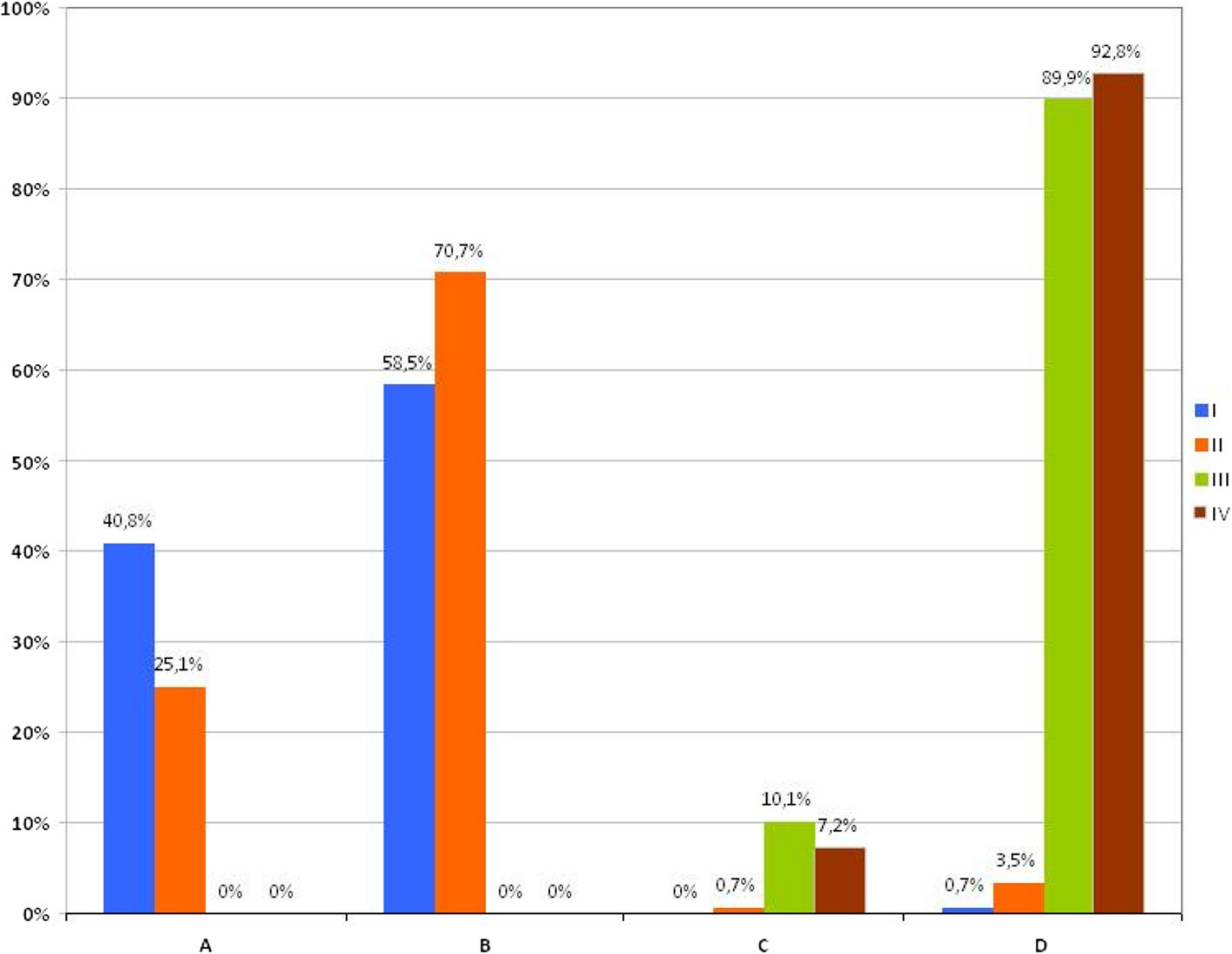
**Distribution of patients with COPD at baseline according to GOLD 2007 and GOLD 2013 classification.**
